# Examining the moment-to-moment association between drinking and problems: The moderating role of emotional functioning

**DOI:** 10.1111/acer.70035

**Published:** 2025-05-08

**Authors:** Noah N. Emery, Hannah A. Carlon, Kyle J. Walters, Sara Mei, Angelina Sung, Angelica DeFalco, Susi Baumgardner, Maggie J. Mataczynski, Mark A. Prince

**Affiliations:** 1 Department of Psychology, Colorado State University, Fort Collins, Colorado, USA; 2 Department of Psychology, Center on Alcohol, Substance Use, and Addictions (CASAA), University of New Mexico, Albuquerque, New Mexico, USA; 3 Department of Individual, Family, and Community Education, University of New Mexico, Albuquerque, New Mexico, USA

**Keywords:** alcohol, negative affect, positive affect, problems, savoring, young adults

## Abstract

**Background:**

Though the link between alcohol use and alcohol-related problems is intuitive, meta-analyses show that drinking explains <20% of the variance in problems. This suggests that other factors play an important role in when and for whom drinking leads to problems. Moreover, most research in this area has relied on either cross-sectional data or macrolongitudinal methods (e.g., 3-month follow-up). This study aimed to examine the moment-to-moment association between drinking and problems, and whether positive and negative affect moderated the strength of this within-person relationship.

**Methods:**

Data were collected using ecological momentary assessment from a college student sample exhibiting hazardous drinking patterns (*n* = 160, observations = 4980). Momentary positive and negative affect were examined as within-person moderators. Person-level averages of positive and negative affect as well as trait savoring (i.e., ability to hold on to positive emotions–shown to be protective against problems) were examined as cross-level moderators of this effect.

**Results:**

Results showed that drinking at the previous moment was strongly associated with reporting more problems at the next moment (*IRR* = 1.74), and higher-than-average levels of negative affect at the previous moment strengthened this moment-to-moment relationship (*IRR* = 1.28). Average positive affect exhibited a cross-level interaction with the moment-to-moment link between drinking and problems (*IRR* = 0.69) such that those with lower-than-average positive affect overall had a stronger association between drinking at the previous moment and the number of problems reported at the next moment. Trait savoring was inversely related to problems (*IRR* = 0.74), and average negative affect was positively associated with problems (*IRR* = 1.27).

**Conclusions:**

This study demonstrates that affect can exacerbate or buffer the moment-to-moment relationship between alcohol use and problems.

## INTRODUCTION

Alcohol use among US college students is associated with a host of negative consequences such as poor academic outcomes, mental and physical health problems, development of alcohol use disorders, and even death (e.g., White & Hingson, 2013). Accordingly, alcohol use and the associated negative consequences among college students have been a major public health concern for decades (Hingson et al., 2009). However, though the link between alcohol use and problems as a result of drinking is intuitive, meta-analyses show that alcohol use (i.e., drinking frequency or quantity) explains <20% of the variance in experiencing problems (Prince et al., 2018). This suggests that other factors beyond levels of alcohol consumption play an important role in for whom and under what conditions drinking is associated with problems. Moreover, most research in this area has relied on either cross-sectional data or macrolongitudinal methods (e.g., baseline and 3-month follow-up; Merrill et al., 2022; Prince et al., 2018). However, interventions are designed to target individual use episodes to produce within-person change in the moment (e.g., harm reduction strategies). As such, one notable gap in knowledge is whether the association between drinking and problems is detectable moment-to-moment in close temporal proximity and under what conditions the link between drinking and problems manifests itself in daily life. Individual drinking episodes, though small in isolation, are necessary building blocks for the development of large patterns in alcohol-related problems reflective of those at risk for alcohol use disorder. Thus, to better inform treatment, there is a need to understand the conditions under which drinking events are linked to problems in the moment, as well as for whom this liability is greatest. This knowledge would improve our understanding of the conditions linked to greater problems that lend themselves to preventing negative consequences and interrupting the development of addiction.

Emotions are basic features of human functioning that influence responses to our environment and help tune decision making, and as such, they play a key role in behavioral health decisions (Gross, 2011). Emotional functioning involves the expression, perception, regulation, differentiation, and awareness of emotions (Milojevich et al., 2021). Emotional functioning figures prominently in almost every contemporary model of alcohol use (Kassel & Veilleux, 2010). These models posit that alcohol use becomes reinforced due to its ability to modulate a person’s affect (Sher & Grekin, 2007) and that addiction pathology is thought to progress as using to regulate affect cycles becomes increasingly reinforced (Koob & Le Moal, 2001). A growing body of literature suggests that, for youth, positive reinforcement (i.e., drinking to increase positive emotions when they are low or to extend their duration) is a leading mechanism facilitating alcohol use (Emery & Simons, 2020; Simons et al., 2014) and alcohol-related problems at the event level (Simons et al., 2014). Specific aspects of positive affective functioning have been identified as potential candidates for altering the link between drinking and problems. Previous research shows momentary *state* and *trait* positive affect exhibit inverse associations with drinking (Emery et al., 2023; Emery & Simons, 2020) and represent an important risk factor for the progression and maintenance of substance use disorders (Emery et al., 2020, 2021).

Negative affect, on the other hand, exhibits a more nuanced relationship with drinking while exhibiting a more consistent relationship with problems. That is, across event-level studies, it is all too common to observe null (Dora et al., 2023; Dvorak & Simons, 2014; Emery et al., 2023), and to a lesser extent positive (Armeli et al., 2008; Emery et al., 2023) or even inverse (Dora et al., 2023; Dvorak & Simons, 2014; Emery et al., 2023; Patrick et al., 2016; Simons et al., 2005), within-person associations between negative affect and drinking. However, negative affect appears to be more consistently linked to more problems at both the within-person (Simons et al., 2005, 2014) and between-person levels (Kuntsche et al., 2005; Simons et al., 2010, 2014). Moreover, high negative affect is associated with more alcohol-related problems, despite lower levels of drinking (e.g., Simons et al., 2014). Taken together, these patterns of effects suggest momentary state and trait positive and negative affect are ideal candidates to help explain when and for whom drinking is associated with problems.

There are also aspects of emotional functioning aside from levels of emotional arousal that may be useful for clarifying the link between alcohol use and problems. One such factor is savoring–the ability to generate, maintain intensity/duration, or enhance positive emotions (Bryant et al., 2011). In previous cross-sectional research, trait savoring has displayed a paradoxical set of associations in which trait savoring exhibits a positive association with drinking and an inverse association with alcohol-related problems (Emery et al., 2024). That is, high savoring is associated with fewer alcohol-related problems, despite higher levels of drinking. This is in line with similar cross-sectional research on cannabis in which savoring moderates the link between use and negative consequences (Luba et al., 2020), as well as preliminary work suggesting savoring interventions reduce cannabis-related problems (Mian et al., 2021; Mian & Earleywine, 2023). Taken together, these series of findings imply a potential attenuating effect of savoring on the link between drinking and problems. However, research has yet to examine whether trait savoring serves as a moderator of the moment-to-moment use–problems relationship.

In order to model moment-to-moment fluctuations in emotional functioning, drinking, and problems over time, advanced data collection methods need to be used that can gather intensive repeated measurements. Ecological momentary assessment (EMA; Stone & Shiffman, 1994) is a technique that uses repeated assessments to capture participants’ emotion and behavior in real time (i.e., event-level) while participants are in their natural environment (i.e., in vivo) through random sampling via mobile devices. EMA offers numerous advantages relative to more commonly used cross-sectional retrospective surveys or several-wave macrolongitudinal designs, especially in terms of appropriately modeling the affect-drinking to problems relationships as dynamically unfolding processes. Given that emotional functioning, drinking events, and experiencing problems are dynamic, fleeting, and variable (e.g., Ebner-Priemer et al., 2009; Larsen & Kasimatis, 1990), it is critical to assess their associations as they are experienced, rather than retrospectively (e.g., Tennen et al., 2000). Moreover, beyond being a more conceptually consistent form of measurement, EMA enhances the generalizability of findings by reducing recall bias that can impact the associations of interest such as mood-congruent memory bias (Bower, 1981; Snyder & White, 1982) and evidence that people often struggle to assign causes to their behavior (Mazar & Wood, 2022; Nisbett & Wilson, 1977). This suggests that modeling these associations in fine-grained temporal proximity is an ideally suited methodological and analytical strategy. Also, research suggests that drinking associations are discordant across levels of analysis (i.e., timescale; Emery et al., 2023; Simons et al., 2014) where a directional finding at one level may not be replicated at another (i.e., day-level vs. moment-level; cf. Simpson’s paradox). An advantage of random sampling of assessments is that the within-person estimates are averaged across all contexts and people, making it some of the strongest possible evidence. In addition, random assessments of drinking and problems ensure that variability is captured that could otherwise be missed if assessments were delivered at fixed intervals. However, despite its strengths, this approach has some drawbacks to consider. Random sampling may miss events of interest, and this is especially true when modeling rare events such as drinking and problems. This produces variables that have strong positive skew and are bound by zero, which precludes certain analyses or research questions from being tested, such as the prediction of specific consequences. Nonetheless, leveraging the strengths of EMA allows researchers to better understand the complex, dynamic, and fleeting processes implicated in the link between momentary drinking and consequences.

This study aimed to examine the moment-to-moment association between drinking and problems, and whether within- or between-person indicators of positive and negative emotional functioning moderate the strength of this within-person relationship using EMA data from a large college student sample exhibiting hazardous drinking patterns. We hypothesized that (1) drinking moments would exhibit a positive association with problems, relative to nondrinking moments; (2) positive affect would moderate this effect at both the within- and between-person levels, such that moments and people with lower positive affect would exhibit a stronger link between drinking and problems; (3) negative affect would moderate this effect at both the within- and between-person levels, such that moments and people with higher negative affect would exhibit a stronger link between drinking and problems; and (4) trait savoring would exhibit a cross-level moderating effect on the within-person association between drinking and problems, making the relationship weaker.

## METHOD

### Participants

An initial sample of 340 college students aged 18–24 was recruited and enrolled in a larger EMA study—The Colorado EMA Project (CEMAP). CEMAP was a large-scale EMA study of young adult college students’ mental and behavioral health conducted from January 2022 to December 2022. The inclusion criteria for CEMAP were (1) currently enrolled college students who were (2) aged 18–24. Given the broad inclusion criteria, many of the participants did not drink alcohol or experience problems related to their drinking. As such, we selected an analytic sample reflecting the population of interest—young adults that potentially experience problems related to their drinking. Participants were only included in the present analyses if they reported hazardous alcohol consumption at baseline (i.e., ≥8 on the Alcohol Use Disorder Identification Test [AUDIT]; Saunders et al., 1993) as these participants likely experienced problems as a result of their drinking. Of the original sample, 160 students met the inclusion criteria (70% female, *M_age_* = 19.1, *SD* = 1.5). Eighty-six percent of the participants identified as White, 1% as Native American, 2% as Asian, 2% as Black/African American, 7% as multiracial, and 2% as another race. Fifteen percent identified as Hispanic/Latine.

### Procedure

Participants were recruited through the university research participation pool. Participants attended an in-person initial enrollment session where they completed informed consent, baseline measures, as well as installed and were trained to use an EMA application on their personal phone. EMA training included discussion of the application’s functions, assessment timing, and survey questions. The EMA protocol was delivered using mEMA (ilumivu, 2021), a cross-platform smartphone application (iOS and Android) developed by ilumivu Inc. The EMA program generated prompts for participants to complete four momentary assessments at random times within 3.5-h blocks from 10a.m. to midnight (e.g., 1:30–5:00p.m.) and a morning assessment between 9:30a.m. and noon each day for 14 days. Random assessments presented brief (~2 min) assessments asking for participants to report on their emotions and behavior during a specified period (e.g., past 30 min, since your last survey) and morning assessments inquired about behavior during the waking hours yesterday (i.e., from when they awaken to when they go to sleep). Participants received partial research credit for the initial enrollment session and each assessment they completed. Only data collected from random assessments were included in the present analysis because these were the only surveys that assessed affect and problems, and our aims were momentary in nature. The study was approved by the Colorado State University Institutional Review Board (#2888).

### Baseline and trait measures

*Demographic information* was collected on participants’ sex assigned at birth, age, race, and ethnicity.

*Alcohol use frequency* in the past 90 days was measured with the Alcohol Use Frequency Rating Scale (AUFRS; Simons et al., 2009). AUFRS uses a single item on a 12-point anchored rating scale (0 = *no use*, 1 = *less than once a month, but at least once in the last 90 days*, 3 11 = *more than once a day*) to assess the frequency of alcohol use in the past 90 days. As part of the AUFRS, definitions of standard drinks with accompanying illustrations were provided (one standard drink = 12 oz. beer, 5 oz. wine, or 1.5 oz. liquor). Past 90-day alcohol use frequency at baseline was used for descriptive purposes only.

*Hazardous drinking* in the past 12 months was assessed with the AUDIT (Saunders et al., 1993). The AUDIT consists of 10 items regarding the frequency of use and alcohol-related problems over the past year. Items are scored between 0 and 4 points, giving a total minimum score of 0 and a maximum score of 40. A score of 8 or more is associated with harmful or hazardous drinking, and a score of 15 or more is likely to indicate alcohol use disorder (*α* = 0.76). As part of the AUDIT, definitions of standard drinks with accompanying illustrations were provided (one standard drink = 12 oz. beer, 5 oz. wine, or 1.5 oz. liquor).

*Savoring* was assessed with the Savoring the Moment Subscale of the Savoring Beliefs Inventory (SBI; Bryant, 2003). This subscale measures a person trait ability to hold on to positive emotions and consists of 8-items such as “when something good happens, I can make my enjoyment of it last longer by thinking or doing certain things,” rated on a 7-point scale (1 = *strongly disagree*, 7 = *strongly agree*). Higher scores indicated a greater savoring tendency (*α* = 0.84).

### Ecological momentary assessment measures

*Affect* in the previous 30 min was assessed during random surveys by items from subscales of the PANAS-X (Watson & Clark, 1994) and Larsen and Diener’s affect circumplex model (Larsen & Diener, 1992) on an 11-point scale (0 = *not at all*, 10 = *extremely*). Five items assessed positive affect: happy, excited, calm, relaxed, and grateful. Five items assessed negative affect: sad, anxious, angry, lonely, and stressed. Previous research supports the internal consistency and criterion validity of these affective items assessed using EMA (Emery et al., 2020; Hoeppner et al., 2014). Positive and negative affect items were averaged into moment- and person-average composite scores for their respective valence.

We calculated the reliability of the positive and negative affect scales for this sample at the within- and between-person levels using McDonald’s Omega (McDonald, 2013) following procedures of Geldhof et al. (2014). McDonald’s omega (*ω*) is an index of internal consistency that uses a factor analytic approach to partition the common variance among the items from the unique variance and determines the general factor saturation of the test. It is the ratio of the common variance to the total variance (common and unique; Dunn et al., 2014). Guidelines for *ω* follow those for Cronbach’s alpha. The positive affect scale exhibited acceptable reliability at the within-person level (*ω* = 0.76) and excellent reliability at the between-person level (*ω* = 0.90). The negative affect scale also exhibited acceptable reliability at the within-person level (*ω* = 0.75) and excellent reliability at the between-person level (*ω* = 0.93).

*Alcohol consumption* was assessed during random surveys by a single item regarding the number of standard drinks they consumed over the past 30 min on an 11-point scale (*0* to *10 or more drinks*). Participants were trained on what constitutes standard drinks at baseline as part of the in-person training. In the current model, momentary drinking was a dichotomous variable signifying the presence (1) or absence (0) at that moment to increase interpretability, given the skew of the momentary drinking data and insufficient variability in the count at the high end of the scale (~2.5% of occasions where participants drank more than 2 standard drinks in the past 30 min). This dichotomous momentary indicator was used to create person-level aggregates reflecting the total number of drinking moments over the sampling period and the proportion of drinking moments over the sampling period. The total number of drinking moments was used for descriptive purposes, and the proportion of drinking moments was included as a person-level predictor in the models. Previous research has demonstrated significant correspondence between self-reported alcohol consumption in an EMA study and transdermal alcohol monitoring (Simons et al., 2015), which supports the validity of this measure.

*Problems* were assessed during random surveys with a checklist of 12 problems adapted from the Young Adult Alcohol Consequences Questionnaire (YAACQ; Read et al., 2006)—a well-established measure of alcohol-related problems. This approach is commonly used in EMA research (Simons et al., 2005; Wray et al., 2014). Participants selected any problems they experienced since their last survey and included the following options: (1) got into a fight, (2) did something embarrassing, (3) got injured, (4) felt confused, (5) blacked out, (6) felt sick to your stomach, (7) passed out, (8) did something impulsive, (9) did something you regret, (10) neglected responsibilities, (11) damaged property, and (12) drove intoxicated. Importantly, the stem question does not ask participants to link the problems they are experiencing to their alcohol use. The rationale behind this approach was that any observed prospective association between problems and drinking would reflect change from what would be expected after an average non-drinking moment. In addition, assessing problems without requiring participants to make an attribution about whether the behavior was the result of drinking provides less biased estimates of the magnitude of the effect of drinking on problems (Gmel et al., 2012). Previous research with the YAACQ supports the criterion validity of these items when incorporated into self-report measures (Bravo et al., 2019). A count of the number of problems endorsed at each moment was used in the current model.

### Transparency and openness

We report how we determined our sample size and all data exclusions. Data were analyzed using Stata 18 (StataCorp, 2023). This study’s design and its analysis were not preregistered. No other articles have been published from these data to date.

### Analysis plan and minimum detectable effect sizes

To test the hypothesized moderating effects of affective functioning on the moment-to-moment association between drinking and problems, we estimated two multilevel models with random intercepts and an unstructured variance–covariance matrix using Stata 18 (StataCorp, 2023). The total number of problems since the last survey was the outcome in both models and was specified with a negative binomial reference distribution. The data had a two-level structure in which moments (Level 1 [L1]; within-person) were nested within persons (Level 2 [L2]; between-person) (see Appendix 1). Multilevel models account for the nonindependence of observations that results from the nesting of time-varying observations via momentary random assessments within persons (L1). Slopes were fixed rather than random because the primary aim lies in estimating the overall effect, rather than exploring between-person variability in slope values. Moreover, incorporating random slopes would introduce additional complexity, particularly when between-person differences in slopes are not central to the research question. The models contained within-person positive affect, negative affect, and drinking at the previous moment predicting problems at the next moment (i.e., lagged effects). These indicators were lagged within day to focus on within-day next-moment associations and avoid lengthy between-day lags in analyses. Alcohol-related outcomes have relatively low base rates (Atkins et al., 2013), which produce datasets that are often positively skewed. This issue is further exacerbated by intensive repeated assessments inherent in event-level studies (Atkins et al., 2013). The negative binomial reference distribution was selected because it accommodates positively skewed count outcomes and expects meaningful zeros.

The first model was a main effects only model. The inclusion of interactions between our focal variables and the within-person association between drinking and problems were included in a second model. Significant interaction effects were probed using a marginal effects at representative values approach (Williams, 2012). Marginal effects are inferential summary statistics that provide a measure of the change in the predicted value of the dependent variable for a one-unit change in a moderating variable while holding all other variables constant. Change is measured in the natural units of the dependent variable. In the models presented here, we calculated the marginal effects of how the relationship between drinking at the previous moment and the number of problems at the next moment varies at representative values of the moderator variables. We used the mean and ±1 *SD* as representative values of the moderator variables consistent with conventions often used in a simple slopes approach, as this gives a clinically meaningful depiction of the specific conditions under which the effects of drinking on problems are likely pronounced that is most comparable to other studies. Importantly, marginal effects quantify the difference in the association (i.e., slopes) that drinking at the previous moment vs. not has with problems at the next moment at the specified representative values of the moderator (i.e., test of equivalence in slopes). Put plainly, this statistic represents a difference in the number of problems reported at the next moment between drinking and nondrinking at the previous moment at specific values of the moderator. Given some have suggested that the significance of the product term in nonlinear models is a poor test for determining whether an interaction is present and the significance of the marginal effects at representative values should be used instead (McCabe et al., 2022), we also examined the marginal effects of nonsignificant product terms at ±1 *SD* to ensure that our effects are articulated properly. We omitted tests at the mean because that is equivalent to a main effect (i.e., effect drinking vs. not on problems at mean negative affect).

L1 focal predictors were within-person previous moment positive affect, negative affect, and alcohol use. In addition to the focal predictors at L1, six day-of-the-week indicators (i.e., dummy coded day of the week with Sunday as the reference group) and day in the study were included as covariates. The inclusion of day of the week addresses daily variation in mood and reduces potential serial auto-correlation across days (Mohr et al., 2001). The inclusion of the number of days since initiating the study adjusts for change over time not associated with the focal predictors. L2 focal predictors were between-person aggregates of the momentary affect and drinking indicators and trait savoring assessed at baseline. L2 covariate was sex assigned at birth, given this is a likely source of differences in problems (Prince et al., 2018; Read et al., 2016). L1 variables were centered within-persons by subtracting person-averages from momentary values (i.e., person-centered). L2 variables were centered by subtracting the overall sample averages from person-level averages (i.e., grand-mean centered). In this context, person-centered variables reflect moment-to-moment deviations from a person’s average level, and grand-mean-centered variables reflect person deviations from the overall average for the sample.

Distinctions between person-average affect and drinking (e.g., dispositional/trait positive affect) and momentary states (e.g., a moment of high positive affect) capitalized on the full complement of the EMA data by distinguishing dynamic, within-person changes in momentary affect and alcohol use from between-person, individual differences in typical (i.e., dispositional/trait) affect and drinking levels. Importantly, EMA data also provide the advantage of temporal specificity. Affect and drinking were expected to vary within- person over time (L1), but also on average from person to person (L2). The inclusion of momentary and average indicators allowed for the isolation of the within-person associations of affect and drinking fluctuations with problems from between-person associations. An intercept-only model (without any predictors) estimated intraclass correlations (i.e., variability in focal variables attributed to between-person effects relative to within-person influences). We did not correct for multiple comparisons in our models following the guidance of Rothman (1990). Instead, describing what tests of significance were performed, and why, is suggested as best practice because corrections protect against Type I errors but also risk creating Type II errors in which important differences might be deemed nonsignificant (Leek et al., 2017; Rothman, 1990).

To determine the minimum detectable effect size from the number of observations in our data, we conducted a series of computer simulations using the Monte Carlo feature of Mplus 8.5 (Muthén & Muthén, 2017). Consistent with previous research, 50% of the variance in affect was specified at the within-person level, with the remaining variance specified at the between-person level (e.g., Emery & Simons, 2020). The focal effects of interest in this study are the interactions between affect indicators and drinking on problems. Unfortunately, previous research has not examined these associations using multilevel analyses. Thus, we conducted multiple Monte Carlo simulations with effect sizes ranging from *β* = 0.05 to *β* = 0.35 for both within- and between-level associations, as well as cross-level interactions. Results using 10,000 replications indicated a sample of 160 individuals with 31 observations each (4960 total observations) would be sufficiently powered (i.e., power ≥ 0.80) to detect within-person effects of *β* = 0.05 or higher, between-person effects of *β* = 0.17 or higher, and cross-level interaction effects of *β* = 0.23 or higher. It is important to note that the final models have more observations and a series of covariates, which will account for additional residual variance not estimated in this model, effectively increasing power above what was seen here. Accordingly, we were adequately powered to detect any clinically meaningful effects found in these models.

## RESULTS

### Descriptive statistics

Compliance with random surveys was calculated by dividing the total number completed by all participants by the total number completed plus missed random surveys. There were 7513 completed random surveys and 8960 combined completed + missed random surveys; thus, compliance was 83.9%. On average, participants completed 46.9 (*SD* = 9.79) random EMA surveys. Participants reported drinking on 24% of the study days and drinking an average of 2.47 (*SD* = 1.53) drinks per drinking day. Participants endorsed at least one problem on 28% of study days and 30% of drinking days. Participants endorsed drinking on 7% of surveys (*SD* = 7.66, range: 0%–48.9%) and reported an average of 1.36 problems per problem moment (*SD* = 0.45, range: 1–3.25). Participants’ mean AUDIT score at baseline was 11.84 (*SD* = 4.08, range: 8–28), indicating hazardous alcohol consumption among the participants. Thirty-three participants (~16%) had AUDIT scores of 15 or greater indicating moderate–severe alcohol use disorder (Kiluk et al., 2018). Sixty-seven percent of participants reported drinking at least once a week in the previous 90 days at baseline. Descriptive statistics for study variables are presented in Table 1.

Positive affect, negative affect, alcohol use, and problems varied across persons and moments. The intraclass correlations were 0.39 for positive affect and 0.47 for negative affect. This indicates that 39% of the variance in positive affect was due to between-person factors, and the remaining 61% was due to moment-to-moment within-person fluctuations. For negative affect, 47% of the variance was due to between-person differences, whereas 53% was due to within-person moment-to-moment variability. Not surprisingly, alcohol use and problems also varied between- and within-persons but exhibited a pattern favoring within-person fluctuations. Specifically, the intraclass correlations were 0.08 for alcohol use and 0.17 for problems. This denotes that between 92% and 83% of the variance in alcohol use and problems was at the within-person level. These findings support the stance that these are time-varying constructs appropriate for event-level methods and analyses.

### Multilevel negative binomial regression models of momentary problems

Prior to examining the moderating effects of affective functioning on the moment-to-moment association between drinking and problems, we first estimated a MLM containing only main effects of focal variables and hypothesized covariates (i.e., day of the week, day in the study). This allowed us to test the hypothesized association between the focal predictors and problems before testing the interactive effects. We then added interactions with drinking at the previous moment.

### Main effects model

At the within-person level (L1), previous moment alcohol use exhibited a strong positive association with problems at the next moment (*IRR* = 1.74, *p* = 0.006). This means drinking moments were associated with a 74% increase in problems at the next moment compared to nondrinking moments. Neither positive nor negative affect at the previous moment was associated with problems at the next moment (*p*s > 0.534). At the between-person level (L2), person-average negative affect was positively (*IRR* = 1.27, *p* = 0.002), and trait savoring was inversely (*IRR* = 0.74, *p* = 0.041), associated with experiencing more problems during the sampling period. Person-average positive affect, sex assigned at birth, and proportion of drinking moments were not related to experiencing problems during the sampling period. See Table 2 for full model estimates.

### Moderation model

To test the hypothesized moderating effects of affective functioning, we modeled the interaction of drinking at the previous moment with our L1 focal variables (i.e., previous moment negative affect, previous moment positive affect) at the within-person level (L1) and cross-level interactions between previous moment drinking and our L2 focal variables (i.e., person-average negative affect, person-average positive affect, trait savoring) on the number of problems experienced at the next moment. Consistent with our hypotheses, at the within-person level (L1), previous moment negative affect moderated the within-person association between drinking and problems (*IRR* = 1.28, *p* = 0.033; Figure 1) such that at higher-than-average negative affect during the previous moment, drinking was associated with an increased number of problems at the next moment compared to nondrinking moments (+1 *SD*: *b* = 0.28, *p* = 0.027, 95% CI: [0.03, 0.53]), and this effect was attenuated as the level of negative affect decreased (*M*: *b* = 0.14, *p* = 0.051, 95% CI: [0.00, 0.29]; −1 *SD*: *b* = 0.05, *p* = 0.474, 95% CI: [−0.08, 0.17]). That is, the slopes between drinking and not drinking at the previous moment were statistically different from each other at high and, to a lesser extent, mean levels of negative affect while no significant difference was observed at low levels of negative affect. Specifically, the predictive margin of problems was significantly higher following drinking moments than following non-drinking moments when momentary negative affect was at or + 1 *SD* above a person’s mean level. Contrary to our hypotheses, previous moment positive affect did not moderate the moment-to-moment association between drinking and problems, compared to nondrinking moments (product term: *p* = 0.848; +1 *SD*: *b* = 0.18, *p* = 0.067, 95% CI: [−0.01, 0.37]; −1 *SD*: *b* = 0.16, *p* = 0.159, 95% CI: [−0.06, 0.38]), meaning that no statistically significant difference in slopes between drinking and not drinking at the previous moment was observed at any representative value of positive affect at the previous moment. In other words, across all levels of momentary positive affect, there was no difference in the predictive margins of problems following drinking vs. nondrinking moments.

At the between-person level (L2), person-average negative affect did not significantly moderate the moment-to-moment association between drinking and problems compared to nondrinking moments (product term: *p* = 0.721; +1 *SD*: *b* = 0.23, *p* = 0.057, 95% CI: [−0.01, 0.47]; −1 *SD*: *b* = 0.09, *p* = 0.276, 95% CI: [−0.07, 0.25]). That is, no statistically significant difference in slopes between drinking and not drinking at the previous moment was observed at any representative value of person-average negative affect. Person-average positive affect moderated the within-person association between drinking and problems (i.e., cross-level interaction; *IRR* = 0.69, *p* = 0.042; Figure 2) such that for people with lower-than-average person-average positive affect, drinking was associated with an increased number of problems at the next moment compared to nondrinking moments (−1 *SD*: *b* = 0.29, *p* = 0.037, 95% CI: [0.02, 0.57]), and this effect was attenuated as levels of person-average positive affect increased (*M*: *b* = 0.13, *p* = 0.076, 95% CI: [−0.01, 0.27]; +1 *SD*: *b* = 0.01, *p* = 0.848, 95% CI: [−0.13, 0.15]). This means the slopes between drinking and not drinking at the previous moment were statistically different from each other at low levels of person-average positive affect, while no significant difference was observed at mean or high levels of person-average positive affect. Specifically, at −1 *SD* below the mean of person-average positive affect, drinking moments were followed by 0.28 more problems than nondrinking moments. While trait savoring did not moderate the momentary drinking to problems association (product term: *p* = 0.342; +1 *SD*: *b* = 0.19, *p* = 0.074, 95% CI: [−0.02, 0.39]; −1 *SD*: *b* = 0.14, *p* = 0.262, 95% CI: [−0.10, 0.37]), it did exhibit an inverse main effect on problems (*IRR* = 0.72, *p* = 0.030) suggesting those with higher savoring experience fewer problems overall during the sampling period. See Table 3 for full model estimates.

## DISCUSSION

Guided by meta-analyses that suggest factors beyond levels of alcohol consumption play an important role in when and for whom drinking is associated with problems (Prince et al., 2018), along with contemporary models of alcohol use suggesting emotional functioning is a central factor in the escalation of drinking-related problems (Koob & Le Moal, 2001; McCarthy et al., 2010; Kassel & Veilleux, 2010), we aimed to characterize the moment-to-moment link between drinking and experiencing problems using EMA and examine whether within- or between-person indicators of emotional functioning moderated the strength of this association. Our findings suggest that drinking when experiencing increased negative affect strengthens the momentary link between drinking and problems, and individuals with lower positive affect on average have a stronger momentary link between drinking and problems. This pattern of results adds to the literature by clarifying the role of negative affect in alcohol use and connecting a person’s average level of positive affect to use and problems in young people. Moreover, the results suggest that an individual’s ability to savor positive affect may be protective against problems. We discuss each finding below in relation to the current literature.

The moderating effect of negative affect at the previous moment is consistent with previous work detailing negative affect’s relationship with alcohol-related problems (Simons et al., 2005, 2014). This study enhances our understanding by adding fine-grained evidence to this body of literature, underscoring that negative affect is linked to more alcohol-related problems on a moment-to-moment basis, rather than solely at a trait level, and that momentary negative affect increases the strength of the prospective momentary association between drinking and problems. Moreover, negative affect exhibited a weak inverse within-person and null between-person bivariate correlation with drinking. These patterns of results reinforce previous work suggesting negative affect is more consistently linked to problems, rather than drinking at both the within-person (Simons et al., 2005, 2014) and between-person levels (Kuntsche et al., 2005; Simons et al., 2010, 2014). Interestingly, despite this empirical evidence, this pattern of associations is not explicitly proposed by any model of affect-motivated drinking (e.g., Kassel & Veilleux, 2010; Sher & Grekin, 2007). The observed relationship may represent the synergistic effects of alcohol and negative affect on cognitive and decision-making processes (e.g., Cyders & Smith, 2008; Miller & Fillmore, 2014; Steele & Josephs, 1990). That is, alcohol consumption and negative affect have been shown to attenuate the top–down information and decision-making processes, resulting in behavioral disinhibition. Thus, impaired cognitive and inhibitory control processes when intoxicated while experiencing heightened negative affect may undermine individuals’ abilities to effectively monitor how their behavior may contribute to problematic outcomes in the moment. Taken together, this set of findings suggests that individuals who experience disproportionate alcohol-related consequences may benefit from interventions that equip them with a range of specific coping skills for managing negative emotions. Furthermore, protective behavioral strategies (PBS) encompass actions that individuals adopt to avert negative consequences linked to alcohol and/or to reduce their alcohol consumption (Martens et al., 2004; Prince et al., 2013; Sugarman & Carey, 2007). Subsequent research could aim to identify strategies that are most effective for young adults when confronted with immediate negative affect or explore new PBS that are designed to be particularly effective at mitigating alcohol-related consequences within a negative affect context. Encouraging young people to use PBS that are more feasible and efficacious at preventing consequences in the context of negative affect could help alleviate the burden of alcohol-related consequences in this population.

The results from the present study also add to the emerging line of studies that implicate low positive affect in the development of heavy alcohol use (Emery et al., 2023; Emery & Simons, 2020), maintenance of substance use disorder (Emery et al., 2020), interference with substance use treatment (Emery et al., 2021), and now strengthening the momentary link between drinking and problems among young people. While negative affect has long stood as a treatment target for problematic alcohol use, the present study’s results additionally suggest that a *lack* of trait-level positive affect is another important variable to consider when aiming to reduce the consequences associated with drinking. Extant research has documented that positive and negative affect are not opposite ends of the same spectrum; rather, they are qualitatively separate phenomena that influence behavior idiosyncratically (Fredrickson, 2001). In fact, these affective states have even been shown to differentially influence how quality-of-life improves for individuals during and after treatment for alcohol use disorder (Carlon et al., 2022). The broaden-and-build theory of positive emotions proposes that experiencing positive affect uniquely facilitates the expansion of one’s behavioral repertoire, fostering new thoughts and actions and setting the stage for an upward trajectory toward a more enriching life (Fredrickson, 2001, 2013). This theory might provide a framework for interpreting the finding that participants with lower trait positive affect demonstrated a more pronounced association between alcohol use and problems. It is plausible that the broadening effect of regularly experiencing positive affect enhances one’s behavioral flexibility and potentially leads to a higher employment of PBS, thus acting as a safeguard against alcohol-related consequences. As such, individuals with higher trait positive affect may be more inclined to use PBS effectively due to the cognitive and behavioral expansion facilitated by positive emotions. Future research might test the applicability of the broaden-and-build theory within this context. Further, these findings imply that interventions geared toward young individuals facing alcohol-related consequences should not only focus on teaching coping mechanisms for moment-to-moment negative affect but also aim to cultivate and amplify trait positive affect. This holistic approach has the potential to yield more effective results at preventing alcohol consequences. Indeed, multifaceted interventions aimed at leveraging positive affect for individuals who use substances or are in recovery have indicated promising results in terms of feasibility, acceptability, and efficacy (e.g., Hoeppner et al., 2019; Krentzman et al., 2015).

The main effect of trait savoring suggests that individuals high in savoring positive emotions are protected from problems, and those low in savoring are at greater risk for experiencing negative consequences, regardless of their drinking patterns, replicating previous cross-sectional research (Emery et al., 2024). This finding carries significance for strategies aimed at reducing alcohol-related harm. Drawing upon the broaden-and-build theory, this result suggests that young people capable of prolonging the experience of positive emotions are inherently better positioned to avoid negative consequences, as the broadening effect of experiencing positive emotions would last longer. Moreover, prior research on savoringbased interventions for people who use substances or are in addiction recovery has yielded promising outcomes regarding safer substance use (e.g., lower substance use, more negative substance use expectancies; Mian & Earleywine, 2023) and heightening positive affect (i.e., higher in-the-moment happiness; Hoeppner et al., 2019). Future studies might delve deeper into the interplay between affect, savoring, and the utilization of PBS. Such research could determine savoring’s potential as a tool for alleviating consequences that some young people experience surrounding their drinking, regardless of how much alcohol they consume.

The results should be interpreted in the context of several limitations. First, the sample for this study was primarily composed of White female college students; thus, generalization to other populations should be done with caution. This racial and sex composition is likely due to the characteristics of the university and community in Northern Colorado where the sample was recruited. It would be of value to evaluate the extent to which these patterns of findings replicate in other sample types (e.g., in treatment-seeking samples of older adults) or if these associations vary as a function of other identity characteristics. The current analyses focused on hazardous drinking to ensure the outcome of interest was likely to be observed for most participants during the sampling period. Future research might benefit from research that models how these associations might differ across lower risk levels. We modeled drinking as a binary variable given variability in momentary drinking quantity was limited above two standard drinks. This means modeling effects of drinking quantity was not possible and these effects might differ when examined as a function of drinking quantity. Our analytic approach was rigorous, and we controlled for contextual factors of import to longitudinal data. However, MLM does not allow for the incorporation of residual autocorrelations, which is a potential limitation. While the temporal ordering is a significant strength compared to cross-sectional research, it does not equate to causality as there is not an experimental manipulation. Yet, as with all research, there is a trade-off between experimenter control and external validity, and the current methodology capitalized on external validity by examining effects in context with temporal precedence between many variables of interest. That said, some of our variables were measured at the same moment (e.g., drinking and negative affect), which places some limits on inferences about the directionality of the interacting predictors. Our problems question did not ask participants to assign cause to options listed, which is a strength given this permits the models to test if drinking was prospectively associated with more problems at the next moment than would be expected after an average nondrinking moment. Yet, some specific problems on the list are potentially more likely to result from drinking (e.g., blacked out, drove intoxicated). The momentary drinking prompt assessed a limited window (i.e., past 30 min), and it is possible that participants drank at times not captured by the random surveys. This could have influenced the results in unknown ways. To speculate, if it is the case that some “nondrinking” assessments may have actually included unmeasured drinking events, there is no reason to believe it would change the direction of the effects observed here. Thus, the observed effects may have underestimated the magnitude of the true effect of drinking on problems, making our estimate likely conservative. Also, some alcohol-related problems are often delayed weeks or months, and our focus on momentary associations does not capture these. Drinking is often a social behavior, and social setting could have influenced the associations observed here in unknown ways. That said, random assessments measured both drinking and affect across multiple social settings (e.g., alone, with friends) and the findings presented here represent the associations averaged across all the possible social settings in which a survey was answered. Lastly, the models estimated here preclude tests of time series and did not control for the potential use of other substances.

Taken together, results from this EMA study found that drinking when experiencing momentary negative affect contributes to a stronger link between alcohol use and problems. Lower trait positive affect also strengthens this link, and a broader capacity to savor emotions is associated with fewer problems overall regardless of alcohol use frequency. These results add to the growing body of literature that underscores the pivotal role of emotional functioning as a significant determinant of for whom and under what conditions young people experience consequences after alcohol use.

## Figures and Tables

**FIGURE 1:**
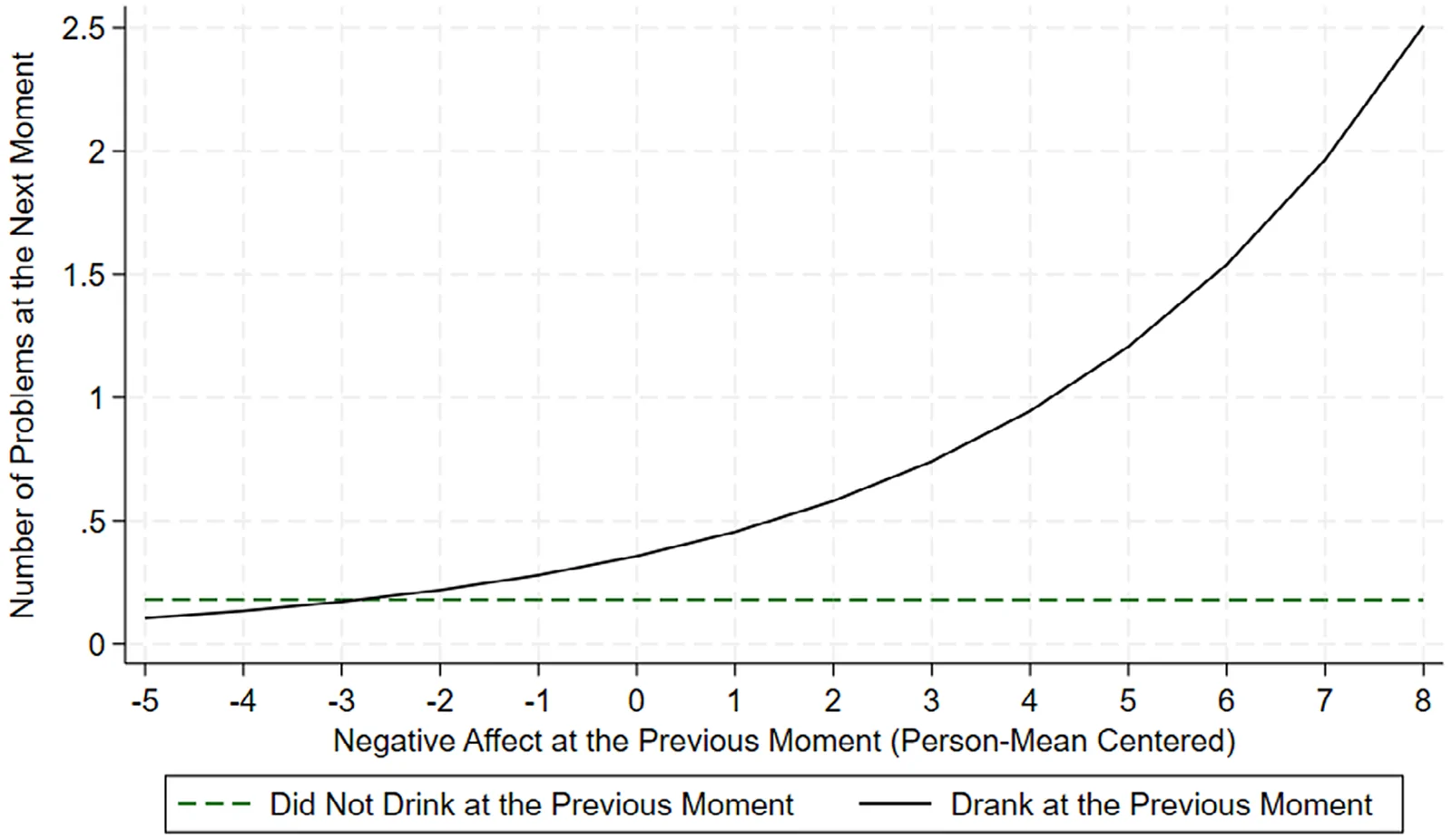
Within-person marginal effects of drinking at the previous moment and previous moment negative affect predicting next-moment problems.

**FIGURE 2:**
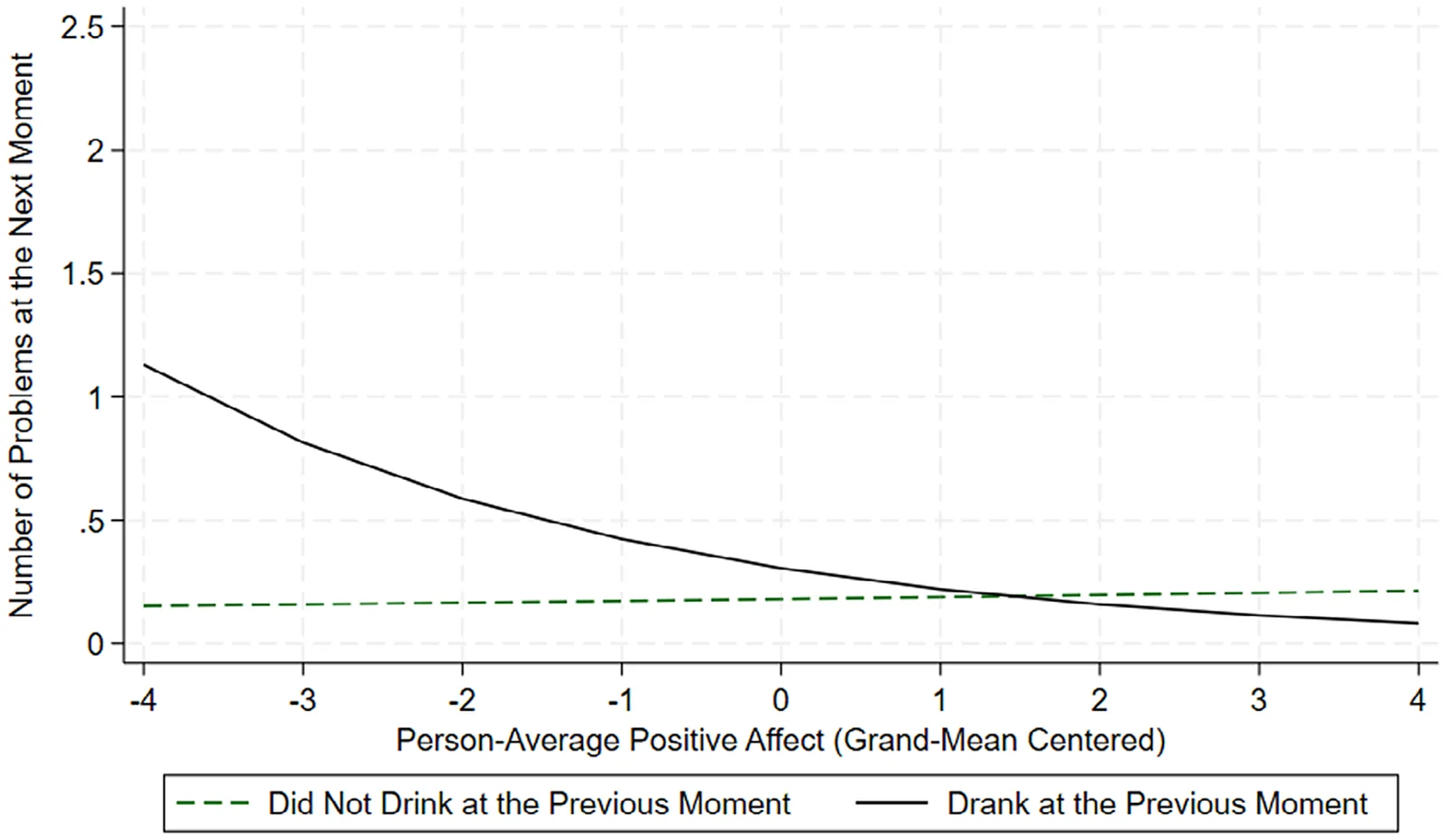
Cross-level marginal effects of drinking at the previous moment x person-average positive affect predicting next-moment problems.

**TABLE 1:** Correlation matrix and descriptive statistics.

Variable	*M*	(SD)	2	3	4	5	6	7	8	9
1. Sex			0.24**	−0.22**	−0.05	−0.07	−0.16*	0.14	0.04	−0.01
2. Age	19.1	1.5		−0.22**	−0.22**	−0.03	−0.13	0.11	−0.01	0.01
3. Negative affect	2.5	1.4			−0.16	0.11	0.30***	−0.26**	0.09	−0.05
4. Positive affect	5.3	1.2		−0.32***		−0.05	−0.01	0.06	−0.02	−0.01
5. Drinking	3.5	3.7		−0.06***	0.06***		0.08	−0.07	0.36***	0.43***
6. Problems	8.7	12.1		0.11***	−0.05***	0.08***		−0.18*	0.09	−0.01
7. Savoring	4.0	0.9							−0.10	−0.06
8. AUDIT	11.8	4.1								0.41***
9. Baseline alcohol use	3.6	1.5								

*Note*: Means and SDs are either person-level aggregates over the sampling period or baseline measures. To the left of the diagonal are the within-person level correlations and to the right are the between-person level correlations. *Within-person note*. Observations 4980 person-moments. Drinking = drank in past 30 min (yes = 1, no = 0). Problems = number of problems since the last survey. Positive and negative affect = mean in past 30 min. *Between-person note. N* = 160. Sex (male = 0, female = 1). Drinking = total number of drinking moments over the sampling period. Problems = total number of problems over the sampling period. Positive and negative affect = person-average aggregates over the sampling period. Baseline alcohol use = past 90 days alcohol use frequency.

* *p* < 0.05,

** *p* < 0.01,

*** *p* < 0.001.

**TABLE 2:** Multilevel main effect model of problems.

	IRR	SE	*p*-value	*95% CI*
Within-person (L1; Time-varying)				
Monday	0.89	0.15	0.489	0.64, 1.24
Tuesday	0.91	0.15	0.568	0.65, 1.27
Wednesday	0.96	0.16	0.818	0.70, 1.33
Thursday	1.03	0.17	0.856	0.75, 1.42
Friday	0.72	0.13	0.056	0.51, 1.01
Saturday	0.89	0.15	0.485	0.63, 1.25
Lagged alcohol use	**1.74**	**0.35**	**0.006**	1.17, 2.59
Lagged negative affect	1.02	0.03	0.534	0.96, 1.08
Lagged positive affect	0.99	0.03	0.751	0.94, 1.05
Day in the Study	**0.95**	**0.01**	**<0.001**	0.93, 0.97

Between-person (L2; Time-invariant)				
Person-average negative affect	**1.27**	**0.10**	**0.002**	1.09, 1.47
Person-average positive affect	1.03	0.09	0.751	0.87, 1.22
Trait savoring	**0.74**	**0.11**	**0.041**	0.55, 0.99
Sex	0.87	0.21	0.554	0.55, 1.39
Proportion of drinking moments	0.99	1.32	0.993	0.07, 13.60

*Note*: *N* = 160. Level 1 observations = 4980 person-momentsLevel 1 variables were person-mean centered and Level 2 variables were grand-mean centered. Sex (female = 0, male = 1). Lagged alcohol use (yes = 1, no = 0). Proportion of drinking moments = proportion of drinking moments over the sampling period. Sunday was the reference group for day-of-the-week indicators. Bold denotes significance.

Abbreviation: IRR, incidence rate ratio.

**TABLE 3:** Multilevel interaction model of problems.

	*IRR*	SE	*p*-value	*95% CI*
Within-person (L1; Time-varying)				
Lagged alcohol use	1.60	0.42	0.074	0.96, 2.67
Lagged negative affect	1.00	0.03	0.984	0.94, 1.06
Lagged positive affect	0.99	0.03	0.638	0.93, 1.05
Lagged alcohol use × Lagged negative affect	**1.28**	**0.15**	**0.033**	1.02, 1.60
Lagged alcohol use × Lagged positive affect	1.03	0.13	0.848	0.79, 1.33
Day in the study	**0.94**	**0.01**	**<0.001**	0.92, 0.96

Cross-level interactions				
Lagged alcohol use × Person-average negative affect	1.05	0.15	0.721	0.80, 1.39
Lagged alcohol use × Person-average positive affect	**0.69**	**0.13**	**0.042**	0.48, 0.99
Lagged alcohol use × Trait savoring	1.27	0.32	0.342	0.78, 2.08

Between-person (L2; Time-invariant)				
Person-average negative affect	**1.27**	**0.10**	**0.002**	1.09, 1.47
Person-average positive affect	1.04	0.09	0.619	0.88, 1.24
Trait savoring	**0.72**	**0.11**	**0.031**	0.54, 0.97
Sex	0.87	0.21	0.547	0.54, 1.38
Proportion of drinking moments	0.84	1.13	0.897	0.06, 11.58

*Note*: *N* = 160. Level 1 observations = 4980 person-moments. Level 1 variables were person-mean centered and Level 2 variables were grand-mean centered. Sex (female = 0, male = 1). Lagged alcohol use (yes = 1, no = 0). Proportion of drinking moments = proportion of drinking moments over the sampling period. Six day-of-the-week indicators were included as covariates but were excluded for clarity. Sunday was the reference group for day-of-the-week indicators. None were significant. Bold denotes significance.

Abbreviation: IRR, incidence rate ratio.

## Data Availability

The data that support the findings of this study are available on request from the corresponding author. The data are not publicly available due to privacy or ethical restrictions.

## APPENDIX 1

Between-person reflects any time-invariant construct. Trait signifies constructs that are measured in trait terms (i.e., ‘in general’; trait savoring). Baseline describes time-invariant constructs measured once at baseline (e.g., baseline alcohol use frequency). Person-level denotes aggregates derived from momentary time-varying constructs (i.e., person-average positive affect, person-average negative affect, proportion of drinking moments).
